# MicroRNA expression profile in intrauterine hypoxia-induced pulmonary hypoplasia in rats

**DOI:** 10.3892/etm.2014.1796

**Published:** 2014-06-20

**Authors:** HUIYI HUO, ZIQIANG LUO, MINGJIE WANG, XIAOHE YU, ZHENGCHANG LIAO, XIAOCHENG ZHOU, SHAOJIE YUE

**Affiliations:** 1 Department of Neonatology, Xiangya Hospital, Central South University, Changsha, Hunan 410008, P.R. China; 2 Department of Physiology, Xiangya School of Medicine, Central South University, Changsha, Hunan 410008, P.R. China; 3 Department of Neonatology, The First Hospital of Hunan University of Chinese Medicine, Changsha, Hunan 410007, P.R. China

**Keywords:** hypoxia, lung development, pulmonary hypoplasia, microRNA, microarray

## Abstract

Hypoxia is necessary for fetal development; however, excess hypoxia is detrimental. The mechanisms underlying the effects of hypoxia on lung development remain unclear, although important roles of microRNAs (miRNAs) during lung development have recently been established. However, the effect on lung development at an miRNA expression level, following changes in oxygen tension, have not yet been studied. In the present study, pregnant rats were exposed to a fraction of inspired oxygen of 10.5 or 21% for two days on gestation day 19, following which the body weight, lung wet weight, radial alveolar count (RAC) and mean linear intercept (Lm) of the newborn pups were analyzed on postnatal day 1. To define the role of miRNAs during lung development following intrauterine hypoxia exposure, the miRNA expression pattern was profiled using a miRNA microarray. The newborn rats in the hypoxic group exhibited statistically significant decreases in body weight, lung weight and the RAC, as well as a marked increase in the Lm. A total of 69 miRNAs were identified to have significant changes in expression, including 55 upregulated and 14 downregulated miRNAs. Quantitative polymerase chain reaction was used to validate the microarray results of six selected miRNAs. Therefore, the results indicated that late gestation intrauterine hypoxia exposure may cause lung injury and miRNAs may play important roles in this process.

## Introduction

Environmental factors have an important role during embryonic development. Exposure to environmental risk factors, including hypoxia, prenatal viral infections, drugs, smoking and stress, results in hypoxia and hypoxic lesions prenatally in ~80% and perinatally in 10–20% of cases (1). These early environmental risk factors may affect the structural and/or functional development of the fetus and neonate. In an hypoxic state, the blood flow in the fetus is concentrated to the brain, heart and adrenals, at the expense of the peripheral organs, particularly the lungs (2). A number of studies have demonstrated that hypoxic exposure during development may lead to lung injury; however, the underlying mechanisms are yet to be elucidated (3,4).

MicroRNAs (miRNAs) have been demonstrated to regulate a number of crucial developmental processes in a variety of organs, with an important role during lung morphogenesis recently established (5). However, little is known with regard to how miRNA expression contributes to critical events in intrauterine hypoxia. The development of the fetal respiratory system is a complex process, and pulmonary growth and maturation is carefully timed and regulated. The process begins early in gestation and extends into adulthood, consisting of five developmental phases, including embryonic, pseudoglandular, canalicular, saccular and alveolar (6). Every phase is critical and indispensable. A number of studies have performed miRNA profiling at a variety of time points corresponding to various phases of rat lung development, from which several miRNAs have been identified that exhibited significant changes in expression (7,8). Hypertensive disorders during pregnancy are the most common cause of intrauterine hypoxia, with a morbidity rate of up to 9.4% in China (9). Hypoxia usually occurs after 20 weeks of gestation, during the canalicular phase of lung development. In order to create an equivalent rat model, hypoxia was initiated in the rats on embryonic day 19 (E19) in the present study.

Numerous studies have confirmed that hypoxia represents a serious risk factor for fetal development (4,10). However, the majority of studies have focused on neural development. In addition, several studies have demonstrated that chronic hypoxia leads to fetal organ dysfunction (11,12), although the majority have investigated miRNA expression during normal lung development (7,13,14). Thus, there are a limited number of studies investigating miRNA expression during lung development following exposure to hypoxia, particularly during pregnancy. In the present study, it was hypothesized that intrauterine hypoxia may result in lung injury and changes in the miRNA expression profile.

## Materials and methods

### Animals

All the animals were obtained from the Animal Center of Central South University (Changsha, China). The animal experimental protocols were approved by the Committee on Research Animal Welfare of Central South University (SCXK-XIANG-2009-0004). Female Sprague-Dawley rats were mated overnight at ~6 weeks of age. The presence of a vaginal plug the following day was used to indicate day 0 of gestation. The pregnant animals were divided randomly into two groups (n=4 per group): Normoxic and hypoxic. All the pregnant rats were assessed for food intake and weight gain on a daily basis.

### Maternal hypoxia

On E19, the mice in the hypoxic group were exposed to 10.5% O_2_. The level of oxygen and length of exposure during pregnancy was conducted as previously described (3). All the hypoxic rats were placed together in a plastic exposure chamber inside an infant incubator. The chamber was filled with room air or pure nitrogen (Changsha Lianhu Acetylene Co. Ltd., Changsha, China). The interior of the hypoxic chamber was continuously monitored for nitrogen (CYX-Digital Oxygen Monitor; Shanghai Jiading Xuelian Instrument Co., Shanghai, China), carbon dioxide concentrations (Fyrite Gas Analyzer; Bacharach, Inc., New Kensington, PA, USA), temperature and relative humidity. The oxygen concentration inside the hypoxic chamber was maintained at 10.5±1.0% and the carbon dioxide concentration was <0.5% inside the exposure chambers. Temperature and relative humidity were maintained at 25±1°C and 50–80%, respectively. Hypoxia exposure was maintained for two days. Rats in the normoxic group were exposed to ambient oxygen (21%) instead. All the rats delivered vaginally at ~22 days gestation.

### Body weight and lung wet weight

The body weight of the pups was obtained on postnatal day 1 (P1). At the end of the experiment, the animals were sacrificed with an intraperitoneal injection of 50 mg/kg pentobarbital (CAS 57-33-0; Sigma, St. Louis, MO, USA) and exsanguinated by severing the femoral aorta. All offsprings were numbered and selected randomly from each group (n=8), half of which were males and half were females. Lung specimens were obtained from each group randomly. Lungs were excised via a midline chest wall incision, cleared of all nonpulmonary tissues and weighed using an electronic scale. The lung samples were then frozen in liquid nitrogen and stored at −70°C for further use. The lung wet weight/body weight ratio (LW/BW) was calculated.

### Lung histology

Lung samples from each group were tracheally-perfused and fixed at 24 cm H_2_O pressure for 24 h, with 4% buffered paraformaldehyde (15). The lungs were sectioned into blocks at right angles to the main bronchus for histological analysis. Tissue blocks were embedded in paraffin and sectioned (4 μm thick). Lung samples were stained with hematoxylin and eosin, and examined for any histological changes. Histological analysis was performed by a pathologist blinded to the experimental group.

### Morphometric analysis

Radial alveolar counts (RACs) were analyzed as previously described (16). Between the center of a bronchiole lined by epithelium in one section of the wall and the nearest connective tissue septum or lung pleural surface, a perpendicular line was constructed using image analysis. The number of alveoli dissected by the line was counted. The RAC was measured for every bronchiole on a slide, from which an average radial alveolar count for the slide was calculated.

Mean linear intercepts (Lm) were measured using crossed hairlines of known length (15). In total, 14 consecutive parenchymal fields from each lung were examined at a magnification of ×200 on 4-μm sections obtained from the left lower lobe. All analyses were performed in a blind manner, without knowledge of the experimental group.

### miRNA microarray

Following RNA isolation from the neonatal pup lungs, a miRCURY™ Hy3™/Hy5™ Power labeling kit (Exiqon, Vedbaek, Denmark) was used for miRNA labeling, according to the manufacturer’s instructions. Each sample (1 μg) was 3′-end-labeled with an Hy3^TM^ fluorescent label using T4 RNA ligase. Briefly, RNA in 2.0 μl water was mixed with 1.0 μl calf intestinal alkaline phosphatase (CIP) buffer and CIP (Exiqon). The mixture was incubated for 30 min at 37°C, and the reaction was terminated by incubation for 5 min at 95°C. Next, 3.0 μl labeling buffer, 1.5 μl fluorescent label (Hy3^TM^), 2.0 μl dimethyl sulfoxide and 2.0 μl labeling enzyme were added to the mixture. The labeling reaction was incubated for 1 h at 16°C, and terminated by incubation for 15 min at 65°C.

Following the labeling procedure, the Hy3^TM^-labeled samples were hybridized on a miRCURY^TM^ LNA Array (version 16.0; Exiqon), in accordance with the manufacturer’s instructions. A total of 25 μl hybridization buffer was added to the 25 μl mixture of Hy3^TM^-labeled samples. The samples were denatured for 2 min at 95°C, incubated on ice for 2 min and hybridized to the microarray for 16–20 h at 56°C in a 12-Bay Hybridization System (Nimblegen Systems, Inc., Madison, WI, USA), which provided an active mixing action and constant incubation temperature to improve hybridization uniformity and enhance the signal. Following hybridization, the slides were obtained, washed several times using a wash buffer kit (Exiqon) and dried by centrifugation for 5 min at 400 rpm. The slides were scanned using an Axon GenePix 4000B microarray scanner (Axon Instruments, Foster City, CA, USA).

Scanned images were imported into GenePix Pro 6.0 software (Axon Instruments) for grid alignment and data extraction. Replicated miRNAs were averaged and miRNAs with intensities of ≥50 times in all samples were selected to calculate the normalization factor. Expressed data were normalized using median normalization. Following normalization, differentially expressed miRNAs were identified via fold-change filtering (fold change of >2.0). Hierarchical clustering was then performed using TIGR MeV software (version 4.6).

### Quantitative polymerase chain reaction (qPCR)

*Taq*Man microRNA assays (Applied Biosystems, Foster City, CA, USA) were performed on selected miRNAs, including miR-465^*^, miR-377^*^, miR-327, miR-338, miR-127 and miR-210, in accordance with the manufacturer’s instructions. In brief, total RNA was isolated from the lungs of the rats using a mirVana miRNA Isolation kit (Ambion, Austin, TX, USA). For qPCR with *Taq*Man microRNA assays, 75 ng total RNA was used as template in each reaction with miRNA-specific reverse transcription (RT) primers. The reactions were incubated on ice for 5 min, followed by incubation at 16°C for 30 min, 42°C for 30 min and 85°C for 5 min. For each PCR assay, 1.33 μl RT product was used as a template. PCR assays were incubated at 95°C for 10 min, followed by 35 cycles of 95°C for 15 sec and 60°C for 60 sec. All the PCR assays were run in duplicate. In addition, RT and PCR were performed for 18S in each sample as an endogenous control using *Taq*Man Ribosomal RNA Control Reagents (Applied Biosystems). Data analysis was performed as aforementioned. RT was performed using the poly(T) adaptor, GCGAGCACAGAATTAATACGACTCACTATAGGTTTTTT TTTTTTVN, while qPCR was performed using the universal reverse primer, GCGAGCACAGAATTAATACGACTCAC, and a forward primer with the same sequence as the mature miRNA (mir-465^*^, UGAUCAGUGCCUUUCUGAGUAG; mir-377^*^, AGAGGUUGCCCUUGGUGAAUUC; miR-127, UCGGAUCCGUCUGAGCUUGGCU; miR-210, CUG UGCGUGUGACAGCGGCUGA; miR-327, CCUUGAGGG GCAUGAGGGU; miR-338, UCCAGCAUCAGUGAUUUU GUUGA).

### Statistical analysis

Results are expressed as the mean ± standard deviation, or percentages. Differences between the groups were analyzed using one-way analysis of variance and the χ^2^-test for categorical variables, with SPSS 15.0 statistical software (SPSS, Inc., Chicago, IL, USA). P<0.05 was considered to indicate a statistically significant difference.

## Results

### Body weight and lung wet weight

**Figure 1:**
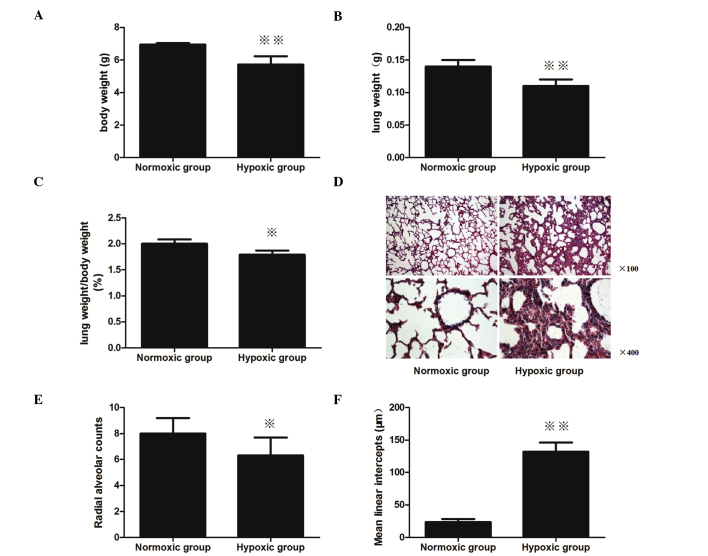
Analysis of lung development. (A) Body weight, (B) lung weight and (C) the lung weight/body weight ratio of the intrauterine hypoxia-treated newborn rats were significantly decreased compared with the intrauterine normoxia-treated rats. (D) Histological observations of the neonatal lungs on day 1 (hematoxylin and eosin stain) in the normoxic and hypoxic groups. The alveolar-like structures in the newborn rat lungs in the normoxic group were irregular and a small number of septa were observed. Compared with the normoxic group, significant lung injury was observed in the animals of the hypoxic group. In addition, the alveolar-like structures were more irregular, the alveolar septum was thick and a small number of red blood cells were present in the alveolar septum and alveolar space. (E) Radial alveolar counts and (F) mean linear intercepts of the intrauterine hypoxia-treated newborn rats were significantly decreased compared with the intrauterine normoxia-treated rats. ^*^P<0.05 and ^**^P<0.01, vs. normoxic group.

Newborn rats at P1 that had been exposed to intrauterine hypoxia from embryonic day 19 (E19) to E20 exhibited a significant body and lung wet weight reduction. The body weight of the hypoxic group pups was significantly reduced compared with the normoxic group (P<0.01; Fig. 1A), as well as the lung wet weight (P<0.01, Fig. 1B). Furthermore, the LW/BW of the hypoxic group pups was also markedly decreased compared with the normoxic pups (P<0.05; Fig. 1C).

### Morphometric analysis

Alveolar-like structures in the newborn rat lungs in the normoxic group on P1 were irregular, and a small number of septa were observed (Fig. 1D). Compared with the normoxic group, significant lung injury was present in the animals in the hypoxic group. In the hypoxic group, the alveolar-like structures were more irregular, the alveolar septum was thick and a small quantity of red blood cells were present in the alveolar septum and alveolar space.

The RAC of the hypoxic-treated lungs was markedly decreased compared with the normoxic-treated lungs on P1 (P<0.05; Fig. 1E). In addition, the Lm was shown to increase in the neonatal rats that had been exposed to intrauterine hypoxia, as compared with the neonatal rats exposed to intrauterine normoxia; the Lm significantly increased from 23.5 to 131.7 μm (P<0.01; Fig. 1F).

### miRNA microarray

**Figure 2:**
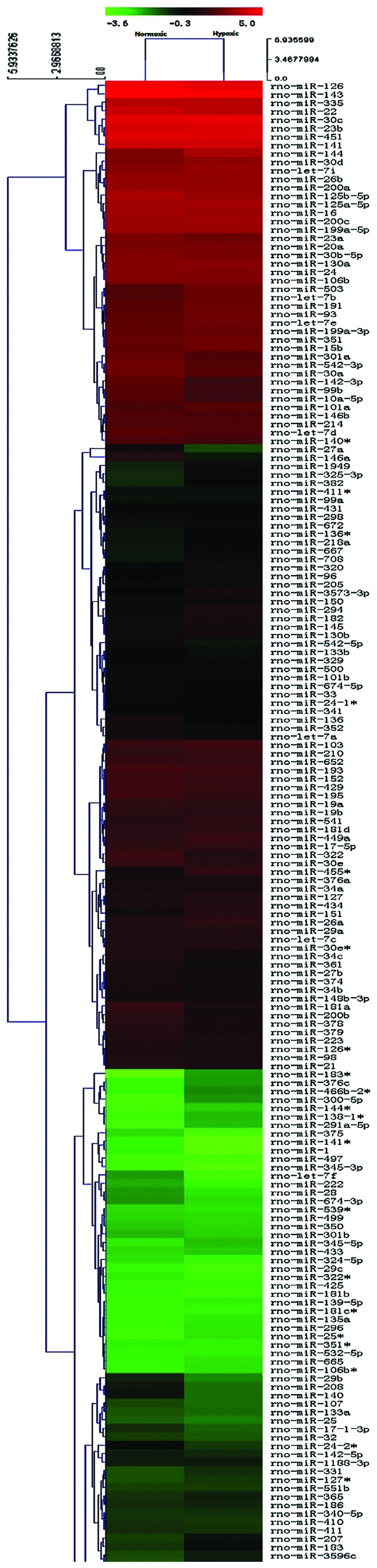
Hierarchical clustering of the two groups (normoxic and hypoxic). Distinguishable microRNA expression profiling is observed. Red indicates high relative expression, green indicates low relative expression and black represents no expression.

The sixth generation of the miRCURY^TM^ LNA Array (version 16.0) was used, which contained >1,891 capture probes, covering all human, mouse and rat miRNAs annotated in miRBase 16.0, as well as all viral miRNAs associated with these species. In addition, this array contained capture probes for 66 novel miRPlus^TM^ human miRNAs. As a result, 69 differentially expressed miRNAs between these two groups passed the fold-change filtering (fold-change of >2.0; Fig. 2), including 55 upregulated miRNAs and 14 downregulated miRNAs (Table I).

### qPCR

**Figure 3:**
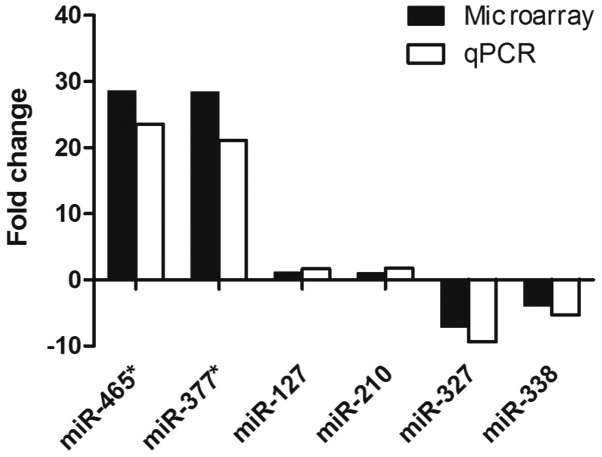
Validation of miRNA microarray results using qPCR. The black bars represent values from microarray analysis, whereas the white bars represent the qPCR data. qPCR, quantitative polymerase chain reaction; miR/miRNA, microRNA.

qPCR was performed to validate the miRNA expression levels in the neonatal rat lungs exposed to intrauterine hypoxia for two days. miRNAs were selected based on high fold changes, their expression in the lungs and functional studies in other systems. miR-465^*^ and miR-377^*^ were selected as the most upregulated miRNAs, while miR-327 and miR-338 exhibited the most downregulated expression. miR-210 was selected as the ‘master hypoxamiR’, since this miRNA has been shown to exhibit high and consistent upregulation under hypoxia in the majority of cell types (17). Furthermore, miR-127 is an important miRNA in late lung development (5,8); thus, was selected. The results from the qPCR analysis revealed that all the selected miRNAs followed the same expression trend observed in the microarray experiment (Fig. 3).

## Discussion

In the present study, intrauterine hypoxia in late gestation was shown to result in lung injury and marked changes in miRNA expression in newborn rats. Hypoxia is required for fetal development; however, excess hypoxia is detrimental. Complications resulting from fetal hypoxia/anoxia are among the top ten causes of fetal mortality (18). As aforementioned, the lung development process begins early in gestation and extends into adulthood, including five developmental phases in rats: Embryonic (E0–E10), pseudoglandular (E11–E18), canalicular (E19–E20), saccular (E21–P3) and alveolar (P4–P21) (6). Each phase is critical and indispensable. Hypoxia can produce temporary dysfunction or permanent injury, depending on the duration, intensity of oxygen deprivation and the age of the fetus (19). However, the mechanisms underlying the effects of hypoxia on lung development remain unclear. Larson and Thurlbeck (20) found that pregnant rats exposed to hypoxia from early gestation (E14) until near term (E21) produced offspring with decreased lung weight, DNA and protein per lung when compared with a normoxic group (20). This long-term intrauterine hypoxia was applied across three periods of lung development. However, the present study investigated whether short-term hypoxic exposure in the more mature lung lead to lung hypoplasia.

In the present study, rats were used as an animal model to investigate hypertensive disorders complicating pregnancy during the canalicular (E19–E20) stage. Newborn rats exposed to intrauterine hypoxia between E19 and E20 exhibited a significant reduction in body and lung wet weight, as well as a marked decrease in the RAC and an increase in the Lm. In addition, the offspring demonstrated fewer and larger alveoli, and the alveolar septum was thicker. The canalicular phase is accompanied by the formation of distal airway bronchioles and proximal to distal epithelial differentiation. Mesenchymal cells begin to develop into chondrocytes, fibroblasts and myofibroblasts (21). A previous study demonstrated that during this stage, hypoxic impairment may irreversibly weaken epithelial growth in the developing lung (22). Any factor affecting normal lung development may disturb the balance between injury and repair, leading to hypoplasia (21). However, the underlying mechanisms are yet to be elucidated.

miRNAs are a large group of regulatory, noncoding, small RNA molecules that are ~22 nucleotides in length (23). Up to a third of human genes involved in the regulation of numerous biological processes, including cellular differentiation, developmental timing, immune responses, nerve system patterning and apoptosis, are regulated by miRNAs (24). Previously, studies have profiled the expression of different miRNAs at various stages of lung development (8), with several studies focusing on the canalicular stage (7,25). However, to the best of our knowledge, no studies have investigated intrauterine hypoxic lung development. In the present study, 69 differentially expressed miRNAs were identified between the hypoxic and normoxic groups, including 55 upregulated miRNAs and 14 downregulated miRNAs, a number of which had not previously been reported.

The expression levels of miR-465^*^ and miR-377^*^ were the most significantly upregulated, while the expression levels of miR-327 and miR-338 were the most downregulated. It has previously been found that miR-465 families are the most abundant X-linked miRNA molecules in newborn mouse ovaries (26). In addition, miR-377 has been shown to be upregulated in diabetic nephropathy and lung tumors (27), and abundantly expressed in transdifferentiated neuronal progenitors (28). Furthermore, miR-327 is upregulated in myocardial microvascular endothelial cells in impaired angiogenesis of type 2 diabetic rats (29), while miR-338 has been previously found to be downregulated in rats with pulmonary fibrosis (30). However, to the best of our knowledge, the present study is the first to investigate these miRNAs during lung development, and in particular, under hypoxic exposure. miR-127 is important during the later stage of fetal lung development (5); however, no statistically significant difference in miR-127 expression was identified between the hypoxic and normoxic groups in the present study. The ‘master hypoxamiR’, miR-210, has been shown to exhibit high and consistent upregulation under hypoxic conditions in the majority of cell types (17); however, the cells used in this study were mature. In the present study, the expression of miR-210 did not change significantly in the developing lung following intrauterine hypoxia exposure when compared with the normoxic group. Studies have found that the increased expression of a number of miRNAs was directly correlated with the downregulation of predicted mRNA targets (31,32). These observations indicated that inhibition of translation without mRNA degradation may be the mechanism of miRNA-mediated gene regulation during lung development. In the present study, more miRNAs were found to be upregulated in the hypoxic group compared with the normoxic group, which may constitute the mechanism underlying lung hypoplasia resulting from intrauterine hypoxia exposure.

In conclusion, the present study demonstrated that intrauterine hypoxia results in lung hypoplasia and marked changes in miRNA expression in newborn rats. However, the systematic profiling of miRNA, mRNA and protein expression levels during intrauterine hypoxic-exposed lung development requires further investigation.

## Figures and Tables

**Table I:** Differentially expressed miRNAs in the hypoxic group compared with the normoxic group^a^.

miRNAs	Fold-change	Expression
rno-miR-465^*^	28.57707510	Upregulated
rno-miR-377^*^	28.38902356	Upregulated
rno-miR-26a^*^	12.85968379	Upregulated
rno-miR-208b-3p	12.24797596	Upregulated
rno-miR-125b-3p	10.71640316	Upregulated
rno-miR-375^*^	10.35918972	Upregulated
rno-miR-3571	8.463210702	Upregulated
rno-miR-192	7.858695652	Upregulated
rno-miR-9^*^	7.144268775	Upregulated
rno-miR-218a-1^*^	7.057959375	Upregulated
rno-miR-291b	5.71541502	Upregulated
rno-miR-124	5.239130435	Upregulated
rno-miR-224^*^	5.080368906	Upregulated
rno-miR-3590-5p	5.000988142	Upregulated
rno-let-7a-2^*^	4.156665469	Upregulated
rno-miR-3572	4.082439300	Upregulated
rno-miR-323	3.969038208	Upregulated
rno-miR-3557-5p	3.572134387	Upregulated
rno-miR-344a	3.798850313	Upregulated
rno-miR-664-2^*^	3.333992095	Upregulated
rno-miR-3594-3p	3.214920949	Upregulated
rno-miR-183^*^	3.162217327	Upregulated
rno-miR-31	2.917243083	Upregulated
rno-miR-3596b	2.857707517	Upregulated
rno-miR-3592	2.843578545	Upregulated
rno-miR-29b-1^*^	2.739856731	Upregulated
rno-miR-374^*^	2.737659550	Upregulated
rno-miR-582^*^	2.729787569	Upregulated
rno-miR-449c-5p	2.698577075	Upregulated
rno-miR-448^*^	2.662863816	Upregulated
rno-miR-2985	2.619565217	Upregulated
rno-miR-296^*^	2.593104963	Upregulated
rno-miR-347	2.578906777	Upregulated
rno-miR-652^*^	2.571936759	Upregulated
rno-miR-551b^*^	2.500494071	Upregulated
rno-miR-331^*^	2.442879000	Upregulated
rno-miR-221^*^	2.429051383	Upregulated
rno-miR-493^*^	2.422838976	Upregulated
rno-miR-16^*^	2.381422925	Upregulated
rno-miR-190b	2.286166008	Upregulated
rno-miR-463	2.266932395	Upregulated
rno-miR-21^*^	2.262351779	Upregulated
rno-miR-300-5p	2.238537549	Upregulated
rno-miR-3577	2.222661397	Upregulated
rno-miR-134^*^	2.177300960	Upregulated
rno-miR-9	2.143280632	Upregulated
rno-miR-202^*^	2.140053487	Upregulated
rno-miR-153^*^	2.139897411	Upregulated
rno-miR-873	2.133579843	Upregulated
rno-miR-1224	2.131559843	Upregulated
rno-miR-208^*^	2.112218594	Upregulated
rno-miR-433^*^	2.102979629	Upregulated
rno-miR-154^*^	2.078332734	Upregulated
rno-miR-1949	2.065437510	Upregulated
rno-miR-455^*^	2.008607060	Upregulated
rno-miR-327	0.142885375	Downregulated
rno-miR-338	0.295624915	Downregulated
rno-miR-222^*^	0.317523057	Downregulated
rno-miR-27a	0.354368736	Downregulated
rno-miR-196a	0.357213439	Downregulated
rno-miR-412	0.357213439	Downregulated
rno-miR-299^*^	0.40824393	Downregulated
rno-miR-133a^*^	0.446516798	Downregulated
rno-miR-23b^*^	0.476284585	Downregulated
rno-miR-27a^*^	0.476284585	Downregulated
rno-miR-29c^*^	0.476284585	Downregulated
rno-miR-504	0.476284585	Downregulated
rno-let-7f	0.490939495	Downregulated
rno-miR-140	0.495260066	Downregulated

a Two-fold downregulated/upregulated miRNAs (arranged from high to low).

miRNA, microRNA.
